# Herpes simplex virus, early neuroimaging markers and incidence of Alzheimer’s disease

**DOI:** 10.1038/s41398-021-01532-2

**Published:** 2021-07-31

**Authors:** Morgane Linard, Marion Baillet, Luc Letenneur, Isabelle Garrigue, Gwenaëlle Catheline, Jean-François Dartigues, Karine Peres, Catherine Helmer

**Affiliations:** 1 University of Bordeaux, Inserm, Bordeaux Population Health Research Center, UMR U1219, F-33000 Bordeaux, France; 2 University of Bordeaux, CNRS-UMR 5234 and CHU Bordeaux, Virology Department, F-33000 Bordeaux, France; 3 EPHE, PSL Research University, Neuroimaging and Daily Life Laboratory, F-33000 Bordeaux, France; 4 University of Bordeaux, CNRS, UMR 5287, Institute of Integrative and Cognitive Neuroscience of Aquitaine, F-33000 Bordeaux, France; 5 Bordeaux University Hospital, Memory Consultation, CMRR, Bordeaux, F-33076 France; 6 Clinical and Epidemiological Research Unit, INSERM CIC1401, F-33000 Bordeaux, France

**Keywords:** Diseases, Pathogenesis

## Abstract

While previous studies suggest the implication of herpes simplex virus (HSV) in the onset of Alzheimer’s disease (AD), no study has investigated its association with early neuroimaging markers of AD. In the Three-City and the AMI cohorts, the associations between HSV infection and (i) hippocampal volume (*n* = 349), (ii) white matter alterations in the parahippocampal cingulum and fornix using diffusion tensor imaging (*n* = 260), and (iii) incidence of AD (*n* = 1599) were assessed according to APOE4 status. Regardless of APOE4 status, infected subjects presented (i) significantly more microstructural alterations of the parahippocampal cingulum and fornix, (ii) lower hippocampal volumes only when their anti-HSV IgG level was in the highest tercile—reflecting possibly more frequent reactivations of the virus (*p* = 0.03 for subjects with a high anti-HSV IgG level while there was no association for all infected subjects, *p* = 0.19), and (iii) had no increased risk of developing AD. Nevertheless, among APOE4 carriers, infected subjects presented lower hippocampal volumes, although not significant (*p* = 0.09), and a two or three times higher risk of developing AD (adjusted Hazard ratio (aHR) = 2.72 [1.07–6.91] *p* = 0.04 for infected subjects and aHR = 3.87 [1.45–10.28] *p* = 0.007 for infected subjects with an anti-HSV IgG level in the highest tercile) while no association was found among APOE4 noncarriers. Our findings support an association between HSV infection and AD and a potential interaction between HSV status and APOE4. This reinforces the need to further investigate the infectious hypothesis of AD, especially the associated susceptibility factors and the possibility of preventive treatments.

## Introduction

Alzheimer’s disease (AD) is a neurodegenerative disease characterized by visible changes within the brain several years before the clinical stage of dementia [1], including hippocampal atrophy and microstructural alterations of white matter (WM) [2–5]. Nevertheless, although the temporality and topography of the clinical and biological markers of AD have been better described, the cause of the disease remains misunderstood.

Several studies have suggested the implication of infectious agents in AD pathophysiology [6], with herpes simplex virus type 1 (HSV-1) being one of the most investigated candidates [7, 8]. In fact, the implication of HSV-1 could potentially explain the location, chronology and the type of damage described in AD. First, HSV-1 is a neurotropic virus with a particular tropism for the temporal cortex, as shown by the localization of lesions in HSV-1 encephalitis and postmortem studies highlighting the presence of HSV DNA in the brains of older adults (reviewed in [9]). HSV-1 also has the ability to move from neurons to neurons, which could explain the progression of lesions to other brain areas [10, 11]. Second, HSV-1, which remains in a latent state throughout life after primary infection, can periodically reactivate during a temporary immune decline. With advancing age, the development of a long-lasting immunosenescence [12, 13] may allow more frequent and/or more intense reactivations of the virus [14], thereby explaining the progressive and relatively late onset of AD. Third, insights from in vitro and animal studies revealed that the inoculation of HSV-1 leads to the development of the main hallmarks of AD, in particular neuro-inflammation, amyloid deposits, and phosphorylated Tau (reviewed in [15, 16]), and that the accumulation of the peptide Aβ may be due to its potential antimicrobial role [6, 17, 18]. Finally, HSV-1 could be linked to genetic risk factors of AD [19, 20], especially APOE4. The existence of such genetic susceptibility factors could explain why, despite a seroprevalence of ~80% in the elderly [21], some infected individuals remain “healthy carriers”, while others develop clinical consequences of the infection.

However, results from epidemiological studies are still inconsistent concerning the association between HSV infection and cognitive decline or dementia (reviewed in [22]) and, if HSV has been related to gray matter volume among AD patients [23–25], no study has investigated its association with earlier neuroimaging markers of AD among the elderly.

In the present study, we aimed to evaluate the association between HSV status and (i) hippocampal volume (HV), (ii) WM alterations in the cingulum and the fornix which are the main afferent and efferent axonal bundles of the hippocampus in two population-based prospective cohorts of older adults. We also complement our previous results [26] in a larger study sample regarding its association with dementia incidence.

## Materials and methods

### Design and participants

The study sample comes from two population-based prospective cohorts: the Three-City study (3C) and the Aging Multidisciplinary Investigation study (AMI). A detailed description of both study protocols was previously published [27, 28]. Briefly, the 3C cohort initially aims to assess the effect of vascular factors on cognitive impairment in three French areas (Bordeaux, Dijon, and Montpellier). In Bordeaux, 2104 participants aged 65 years or older, non-institutionalized, and enrolled from electoral lists were included in 1999–2001. The AMI cohort aims to study health and aging among the elderly population living in rural settings. In 2007–2009, 1002 retired farmworkers or owners from the south-west of France who were aged 65 years or older were enrolled from the reimbursement database of the French Farmer Health Insurance System. Data on sociodemographic status, lifestyle, and health were collected during home visits 2, 4, 7, 10, 12, and 14 years and 2, 4, and 7 years after inclusion, respectively, for 3C and AMI. The Ethical Committee of the University Hospital of Kremlin-Bicêtre and Sud-Mediterranée III for 3C and of Bordeaux for AMI approved the research protocol. Each participant provided signed informed consent.

### Determination of HSV status

HSV serologies were based on blood samples provided at the 4-year follow-up visit for 3C (*n* = 1258) and at inclusion for AMI (*n* = 609) (Supplementary Fig. 1). Two types of anti-HSV antibodies were determined: anti-HSV IgG, reflecting an infection by the virus, and anti-HSV IgM, possibly representing a reactivation of the virus in the weeks before blood sampling. We used LIAISON® HSV-1/2 IgG/IgM tests (Diasorin, Saluggia, Italy—chemiluminescence immunoassay technology) (details in Supplementary Information). Participants were excluded if their serology was inconclusive (*n* = 40) or if they presented IgG- IgM+ status (*n* = 5) reflecting either a primary infection (unlikely given the advanced age of the participants) or IgG false negative or IgM false positive (Supplementary Fig. 1).

### Incidence of AD

In both cohorts, an active screening of cognitive and functional decline was performed at each visit by a trained neuropsychologist. When cognitive impairment was suspected, a second evaluation was performed by a geriatrician or a neurologist to confirm the diagnosis of dementia using the Diagnostic and Statistical Manual of Mental Disorders Fourth Edition criteria [29] and to determine the etiology. Each case of dementia was then reviewed by a committee of experts according to the NINCDS-ADRDA criteria for AD. For statistical analyses of the incidence of AD (including pure AD and mixed dementia), we excluded subjects with prevalent dementia at the time of serology (*n* = 133) and subjects without any follow-up after the time of serology (*n* = 171). Analyses were carried out on 1599 participants (Supplementary Fig. 1). Among them, the participants from 3C were already included in previous published results [26].

### Magnetic resonance imaging acquisition and processing

Magnetic resonance imaging (MRI) was performed using an ACHIEVA 3T scanner (Philips Medical System, Netherlands) (details in Supplementary Information) on a subsample of 238 participants 6 years after the serology in 3C and 200 participants 4 years after the serology in AMI. Among these subjects, 387 had anti-HSV serologies.

3D T1 weighted images were used to segment the hippocampus using FIRST implemented in FMRIB Software Library v5.0 (FSL, [30]). The quality of segmentation was checked manually for each subject with minimal manual editing if necessary. A total of 35 participants had no available measure of the HV and three participants were already demented at the time of the serology, resulting in the inclusion of 349 subjects in this analysis (Supplementary Fig. 1). Right and left HVs were extracted, averaged, and expressed as a percentage of the total intracranial volume (TIV).

WM integrity was assessed using diffusion tensor imaging (DTI). Of 387 participants initially included in the MRI study, 259 participants had available diffusion-weighted images (Supplementary Fig. 1). None of them were demented at the time of the serology. Briefly, diffusion-weighted images (DWI) were co-registered to the reference b0 volume with an affine transformation and were corrected for motion and eddy current distortions. The four successive DWI runs were then averaged and fractional anisotropy (FA), mean diffusivity (MD), and axial and radial diffusivities (AxD, RD) maps were obtained by fitting a tensor model using FMRIB’s Diffusion Toolbox (FDT), part of FSL. FA, MD, AxD, and RD maps were nonlinearly registered to the FMRIB58-FA template and then affine transformed to the MNI152 space using the Tract Based Spatial Statistics pipeline [31]. Mean FA and diffusivity values were extracted on the registered diffusion maps within a study-specific mean WM skeleton mask (threshold: 0.2) using fslmeants function for two axonal bundles: the fornix and the cingulum in the portion running along the hippocampus, as defined in the Johns Hopkins University atlas (stria terminalis of the fornix and hippocampal cingulum). We focused on the fornix and the cingulum as they are the main afferent and efferent axonal bundles of the hippocampus. In consequence, they are considered as particularly interesting biomarkers in the amnesic form of Alzheimer’s disease and several studies have highlighted alterations of white matter microstructure in these bundles from the early stages of the disease [2–5]. FA ranges from 0 to 1 and evaluates the directionality of water diffusion in the brain. In WM, an FA value close to 1 indicates that water molecules preferentially move in one main direction (anisotropic motion), along axonal fibers. MD represents the average amplitude of water diffusion movements measured in the three space directions (*x*,*y*,*z*), AxD the diffusivity in the main direction and RD the average of the diffusivity in the two remaining directions. High FA and low diffusivity values reflect a high level of structural coherence and undamaged axon fiber architecture.

### Statistical analysis

For analyses of HV and WM integrity, we considered only IgG antibodies because small sample sizes prevented us from analyzing IgM. Both the presence and the levels of IgG in terciles were considered. The use of a continuous variable for IgG levels was not possible due to the presence of an upper limit of quantification. Linear regression models adjusted for age at serology, sex, level of education, presence of at least one allele of APOE4, hypertension, diabetes, and cohort were performed. Normality of residuals and homoscedasticity were assessed using a histogram of residuals, a normal quantile-quantile plot of residuals, and a plot of residuals versus predicted values.

For analyses of the incidence of AD, we considered the presence of IgG and IgG levels in terciles. The existence of an interaction with time prevented us from analyzing the IgM status. Cox models, using age as timescale, were performed [32]. The results are presented as the cause-specific hazard ratios (HRs) with their 95% confidence intervals (95% CIs) and *p*-values. Age at dementia onset (AD or other types) was defined as the middle of the time interval between the last visit without dementia and the diagnosis visit. Participants who developed another type of dementia were censored at the age of onset of dementia. Nondemented participants (included the deceased without dementia) were censored at the age of the last visit without dementia. We considered the following confounding factors in addition to age (as timescale): sex, level of education, marital status, presence of at least one allele of APOE4, hypertension, diabetes, hypercholesterolemia, tobacco use, and cohort. The proportional hazards assumption was assessed with Schoenfeld residuals and by testing interactions with time.

For all analyses, we tested the existence of an interaction between APOE4 and HSV status. As we assumed a priori a different impact of HSV infection according to the APOE4 status, analyses were then systematically stratified by APOE4. In the stratified models, due to the small sample sizes, the adjustment variables were restricted to age, sex, and cohort for linear regression models and to age (as a timescale), sex, level of education, and cohort for Cox models. For the same reason, we did not consider the levels of IgG in terciles in the stratified samples.

All statistical tests were two-tailed, and the threshold for statistical significance was 5%. Analyses were performed with SAS software (version 9.4 SAS Institute).

## Results

### Characteristics of participants

The characteristics of participants are presented in Table 1 in the three studied samples: HV (*n* = 349), WM integrity (*n* = 259), and AD incidence (*n* = 1599). In the latter, participants were 77 (±5) years old, 45% were male and 18% were APOE4 carriers. In this sample 83% (*n* = 1327) had anti-HSV IgG and these tended to be more often women, widowed, with a lower level of education, more hypertension, and more often non-smoker (Supplementary Table 1).

**Table 1:** Characteristics of participants included in analyses on hippocampal volume, white matter integrity, and incidence of AD.

	Sample for hippocampal volume analyses (*N* = 349) *N* (%)	Sample for white matter integrity analyses (*N* = 259) *N* (%)	Sample for incidence of AD analyses (*N* = 1599) *N* (%)
Age			
Mean (standard deviation)	74.1 (4.5)	73.9 (4.3)	76.7 (5.3)
Minimum/maximum	65.9/85.3	65.9/85.0	65.7/96.1
Sex			
Male	163 (46.7)	119 (45.9)	716 (44.8)
Level of education			
Elementary school without diploma	70 (20.1)	51 (19.7)	361 (22.6)
Short secondary school	165 (47.3)	118 (45.6)	787 (49.2)
Higher levels	114 (32.7)	90 (34.7)	451 (28.2)
Marital status^a^			
Married	232 (66.7)	174 (67.2)	934 (58.5)
Widowed	79 (22.7)	57 (22.0)	470 (29.4)
Single or divorced or separate	37 (10.6)	28 (10.8)	192 (12.0)
APOE4^a^			
At least one allele	69 (20.8)	51 (20.9)	271 (17.5)
Hypertension^a,b^			
Presence	258 (74.1)	186 (72.1)	1266 (79.3)
Diabetes^a,b^			
Presence	26 (7.4)	17 (6.6)	199 (12.5)
Hypercholesterolemia^a,b^			
Presence	204 (58.5)	155 (59.8)	933 (58.4)
Tobacco consumption^a^			
Former or current smoker	121 (34.7)	91 (35.1)	555 (34.8)
Anti-HSV IgG			
Positive	281 (80.5)	206 (79.5)	1327 (83.0)
Anti-HSV IgM			
Positive	11 (3.2)	9 (3.5)	61 (3.8)

3C and AMI cohorts.

*AD* Alzheimer’s disease.

^a^The number of missing data were 1, 0, and 3 for marital status, 18, 15, and 54 for APOE4, 1, 1, and 3 for hypertension, 0, 0, and 8 for diabetes, 0, 0, and 1 for hypercholesterolemia, and 0, 0, and 6 for tobacco consumption for the samples used for hippocampal volume analyses, white matter integrity analyses and incidence of Alzheimer’s disease analyses, respectively.

^b^Hypertension was defined as taking antihypertensive treatment or having blood pressure ≥140/90 mmHg (vs blood pressure <140/90 mmHg without antihypertensive treatment). Diabetes was defined as taking anti-diabetic treatment or having fasting blood sugar ≥7 mmol/L or ≥11 in case of non-fasting blood sugar (vs fasting blood sugar <7 mmol/L or <6.1 in case of non-fasting blood sugar and without anti-diabetic treatment). Hypercholesterolemia was defined as taking lipid-lowering treatment or having cholesterol level ≥6.2 mmol/L (vs cholesterol level <6.2 mmol/L without lipid-lowering treatment).

### Association between HSV status and HV

Among the available sample, 80.5% (*n* = 281) had anti-HSV IgG. The mean HV expressed as a percentage of the TIV was 0.25 ± 0.03 (Table 2).

**Table 2:** Hippocampal volume and white matter integrity of the parahippocampal cingulum and fornix.

	Total	
	Mean (SD)	Minimum/maximum
Hippocampal and total intracranial volumes (*n* = 349)		
Total intracranial volume in cm^3^	1329.3 (127.4)	1054.9/1758.7
Mean hippocampal volume in cm^3^	3.27 (0.45)	2.05/4.35
Hippocampal volume (% TIV)	0.25 (0.03)	0.15/0.32
DTI parameters (*n* = 259)		
Parahippocampal cingulum		
Fractional anisotropy	0.45 (0.04)	0.34/0.56
Mean diffusivity (in 10^−6^ mm^2^/s)	772.3 (39.8)	687.3/896.2
Axial diffusivity (in 10^−6^ mm^2^/s)	1207.6 (60.7)	1039.7/1367.6
Radial diffusivity (in 10^−6^ mm^2^/s)	554.6 (42.7)	453.5/684.7
Parahippocampal fornix		
Fractional anisotropy	0.52 (0.04)	0.40/0.62
Mean diffusivity (in 10^−6^ mm^2^/s)	808.9 (69.1)	701.3/1148.4
Axial diffusivity (in 10^−6^ mm^2^/s)	1305.5 (81.7)	1144.1/1625.5
Radial diffusivity (in 10^−6^ mm^2^/s)	560.7 (72.2)	430.9/939.6

3C and AMI cohorts.

*TIV* total intracranial volume, *SD* standard deviation, *DTI* diffusion tensor imaging.

**Fig. 1:**
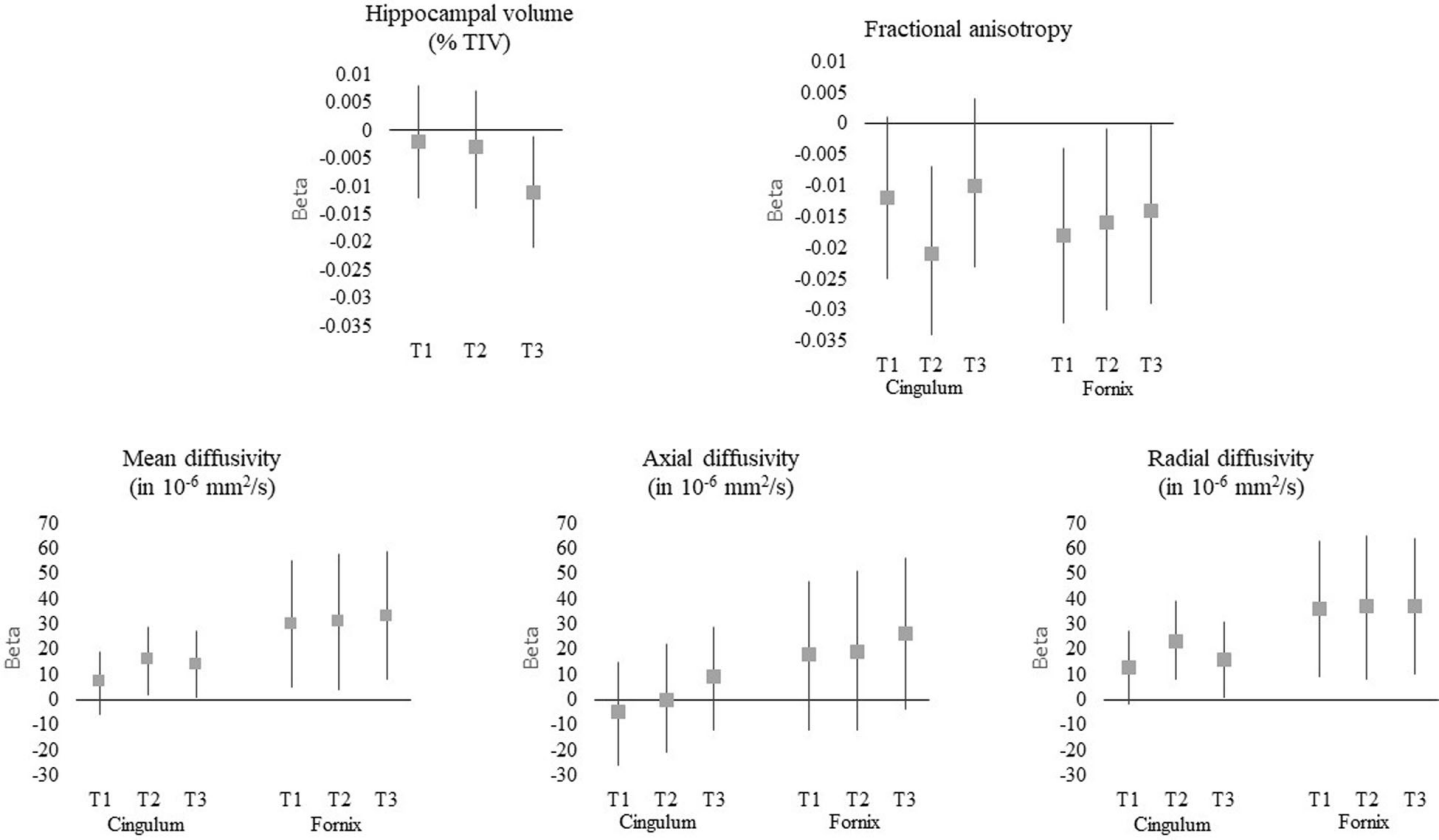
Association between the levels of anti-HSV IgG in terciles and hippocampal volume or white matter integrity. Adjusted linear regression models. 3C and AMI cohorts. T1, T2, and T3: 1st, 2nd, and 3rd terciles of anti-HSV IgG, TIV: total intracranial volume, fornix and cingulum: parahippocampal fornix and cingulum. The bars show the regression coefficients with their 95% confidence interval (reference: uninfected subjects). Linear regression models are adjusted for age at serology, sex, level of education, presence of at least one allele of APOE4, hypertension, diabetes, and cohort.

In non-stratified analyses (Table 3), there was no significant difference in HV between infected and uninfected subjects (*p* = 0.19). Nevertheless, when considering the levels of IgG in terciles, subjects with an IgG level in the highest tercile had a lower HV compared to uninfected subjects (*β* = −0.011 standard deviation = 0.005, *p* = 0.03) (Fig. 1).

**Table 3:** Association between the presence of anti-HSV IgG and hippocampal volume or white matter integrity.

	All subjects, *n* = 330 or 243^a,b^					APOE4-positive subjects, *n* = 69 or 51^a,c^				APOE4-negative subjects, *n* = 262 or 193^a,c^			
	Numbers of IgG negative/positive subjects	*β*	SD	*p*-value	Interaction with APOE4 (*p*-value)	Numbers of IgG negative/positive subjects	*β*	SD	*p*-value	Numbers of IgG negative/positive subjects	*β*	SD	*p*-value
Hippocampal volume (% TIV)	62/268	−0.006	0.004	0.19	0.15	14/55	−0.016	0.009	0.09	48/214	−0.002	0.005	0.71
Parahippocampal cingulum	47/196					11/40				36/157			
Fractional anisotropy		**−0.014**	**0.006**	**0.02**	0.64		−0.014	0.014	0.30		−0.012	0.006	0.05
Mean diffusivity (in 10^−6^ mm^2^/s)		**12**	**6**	**0.04**	0.65		11	12	0.36		10	6	0.09
Axial diffusivity (in 10^−6^ mm^2^/s)		1	9	0.89	0.73		4	19	0.83		−1	10	0.91
Radial diffusivity (in 10^−6^ mm^2^/s)		**17**	**6**	**0.009**	0.72		15	15	0.32		**15**	**7**	**0.01**
Parahippocampal fornix	47/196					11/40				36/157			
Fractional anisotropy		**−0.016**	**0.006**	**0.009**	0.33		−0.025	0.014	0.07		−0.013	0.007	0.06
Mean diffusivity (in 10^−6^ mm^2^/s)		**31**	**11**	**0.005**	0.62		36	19	0.07		**25**	**13**	**0.006**
Axial diffusivity (in 10^−6^ mm^2^/s)		21	13	0.11	0.98		16	24	0.50		16	15	0.28
Radial diffusivity (in 10^−6^ mm^2^/s)		**37**	**12**	**0.002**	0.49		**46**	**22**	**0.04**		**30**	**13**	**0.03**

Adjusted linear regression models. 3C and AMI cohorts.

*TIV* total intracranial volume, *SD* standard deviation.

^a^Number of subjects included in the analysis of hippocampal volume and white matter integrity, respectively (19 and 16 subjects had missing data for APOE4 or hypertension).

^b^Adjusted for age at serology, sex, level of education, presence of at least one allele of APOE4, hypertension, diabetes, and cohort.

^c^Adjusted for age at serology, sex, and cohort.

Bold values are statistically significant results.

The *p*-value of the interaction between the presence of anti-HSV IgG and APOE4 was equal to 0.15 (Table 3). In APOE4 carriers (*n* = 69), the association between the presence of IgG and lower HVs was close to significance (*β* = −0.016 standard deviation = 0.009 *p* = 0.09). No association was observed among APOE4 noncarriers (*n* = 262, *p* = 0.71).

### Association between HSV status and WM microstructural integrity

Among the available sample, 80% (*n* = 206) had anti-HSV IgG. FA, MD, AxD, and RD for the parahippocampal cingulum and fornix are presented in Table 2. Regardless of APOE4 status (Table 3), the presence of anti-HSV IgG was significantly associated with low FA, high MD, and high RD of the parahippocampal cingulum and fornix. No significant associations were observed between the presence of IgG and AxD. Although there were significant associations for several IgG terciles, there were no clear trend for an increase in DTI alterations with increasing IgG levels (Fig. 1).

There were no significant interaction between the presence of anti-HSV IgG and APOE4 for DTI parameters (Table 3, *p*-values ≥ 0.33). There were associations at least borderline significant (*p* ranging from 0.006 to 0.09) between anti-HSV IgG and low FA, high MD, and high RD among APOE4 noncarriers for the parahippocampal cingulum and among both ApoE4 carriers and noncarriers for the parahippocampal fornix.

### Association between HSV status and the incidence of AD

Among the 1599 participants, 83% (*n* = 1327) had anti-HSV IgG. During the follow-up (mean time = 6.8 years, standard deviation 2.6), 293 cases of dementia were diagnosed, including 222 cases of AD, with an incidence rate of 20.6 cases of AD per 1000 person-years. The median time between the serology and diagnosis of AD was 4.3 years (1st quartile = 1.9, 3rd quartile = 6.8).

In non-stratified analyses (Table 4), there was no significant association between HSV status and the incidence of AD. However, the *p*-value of the interaction between the presence of anti-HSV IgG and APOE4 was equal to 0.05 (Table 4). In APOE4 carriers (*n* = 271), the presence of IgG was significantly associated with an increased risk of AD (aHR = 2.72 [1.07–6.91] *p* = 0.04) and subjects with an IgG level in the highest tercile had an increased risk of AD compared to uninfected subjects (aHR = 3.87 [1.45–10.28] *p* = 0.007) or compared to infected subjects with an IgG level in the lowest tercile (aHR = 2.30 [1.08–4.91] *p* = 0.03). In APOE4 noncarriers, no significant association was observed.

**Table 4:** Association between HSV status and the incidence of Alzheimer’s disease—adjusted Cox models.

	All subjects^a^ (*n* = 1528 including 212 cases or *n* = 1269 including 181 cases)^c^				APOE4-positive subjects^b^ (*n* = 271 including 54 cases or *n* = 222 including 49 cases)^d^			APOE4-negative subjects^b^ (*n* = 1274 including 161 cases or *n* = 1062 including 135 cases)^d^		
	aHR	95% CI	*p*-value	Interaction with APOE4 (*p*-value)	aHR	95% CI	*p*-value	aHR	95% CI	*p*-value
Anti-HSV IgG + (vs −)	1.19	0.81–1.77	0.38	**0.05**	**2.72**	**1.07–6.91**	**0.04**	0.92	0.60–1.42	0.72
IgG levels in terciles (in all subjects)^e^			0.37	0.12			**0.02**			0.98
1st tercile (vs negative IgG)	0.99	0.62–1.59	0.98		1.70	0.57–5.05	0.34	0.89	0.53–1.49	0.66
2nd tercile (vs negative IgG)	1.21	0.76–1.92	0.42		2.41	0.85–6.81	0.10	0.93	0.55–1.56	0.78
3rd tercile (vs negative IgG)	1.32	0.87–2.03	0.20		**3.87**	**1.45–10.28**	**0.007**	0.94	0.58–1.51	0.79
IgG levels in terciles (in IgG+ subjects)^e^			0.29	0.42			0.08			0.95
2nd tercile (vs 1st tercile)	1.22	0.81–1.84	0.35		1.43	0.63–3.25	0.40	1.05	0.66–1.69	0.84
3rd tercile (vs 1st tercile)	1.35	0.93–1.97	0.12		**2.30**	**1.08–4.91**	**0.03**	1.07	0.70–1.64	0.75

3C and AMI cohorts.

*aHR* adjusted hazard ratio, *95% CI* 95% confidence interval.

^a^Adjusted for sex, level of education, marital status, presence of at least one allele of APOE4, hypertension, diabetes, hypercholesterolemia, tobacco use, and cohort.

^b^Adjusted for sex, level of education, and cohort.

^c^Number of subjects for analysis in the whole sample or in the subsample of IgG+ subjects, respectively. Among them, 71 and 58 participants had missing data for the adjustment variables, respectively, including 54 and 43 participants with missing data for APOE4.

^d^Number of subjects for analysis in the whole sample or in the subsample of IgG+ subjects, respectively.

^e^IgG levels in the 1st tercile were <17.7, in the 2nd tercile were ≥17.7 and <27.9, and in the 3rd tercile were ≥27.9.

Bold values are statistically significant results.

## Discussion

### Main results

Our findings suggest an increased risk of AD in subjects infected by HSV. Infected subjects with an anti-HSV IgG level in the highest tercile (possibly reflecting more frequent reactivations over time) had lower HV compared to uninfected subjects. Infected subjects presented also more microstructural alterations of the parahippocampal cingulum and fornix regarding FA, MD, and RD. Our results are also in favor of an interaction between being infected with HSV and APOE4 status regarding advanced markers of the disease (HV and then incidence of AD). Among APOE4 carriers, infected subjects presented lower HVs, although not significant, and had a two times higher risk of developing AD and a three times higher risk if their anti-HSV IgG level was in the highest tercile. Among APOE4 noncarriers, no associations were observed between HSV status and HV or incidence of AD.

### Strengths and limitations

Our study has several strengths. The cohorts benefit from a population-based recruitment strategy allowing for the inclusion of participants regardless of their cognitive performance, unlike clinical studies. Nevertheless, some selection bias persists (volunteer bias, acceptance of neuroimaging examination, and blood sampling). As expected, the subjects with brain imaging data were younger, more educated, less often widowed, presented less co-morbidities such as hypertension and diabetes compared to the excluded subjects. It should also be noted that the frequency of APOE4 was slightly increased in these groups (Supplementary Table 2). The HSV serology used has excellent sensitivity and specificity (99 and 97% according to the manufacturer), and the seroprevalence observed in our population was in accordance with previous studies [21]. This serology does not, however, distinguish between HSV-1 and HSV-2 infections, but it seems to be a minor limitation regarding the relatively low prevalence of HSV-2 (≈20%) compared to that of HSV-1 (≈80%) in aged subjects and the tropism of HSV-2 for genital locations that makes it unlikely to be involved in the development of AD. Thus, the detection of both HSV-1 and HSV-2 probably underestimated the observed associations rather than the opposite. Our study has numerous and valuable neuroimaging data on both HV and WM integrity. Therefore, a relatively large number of tests were performed and may have increased the probability of obtaining some statistically significant results purely by chance. The availability of genetic data on APOE4 allowed us to perform stratified analyses, but, due to the small number of APOE4 carriers, analyses were adjusted only for a limited number of potential confounders. Note that, as sensitivity analyses, the stratified models were also carried out with the full set of adjustment variables. The results were unchanged except for two analyses concerning the parahippocampal fornix: the association became non-significant among the APOE4 carriers for RD (*p* = 0.06 vs 0.04) and significant among the APOE4 noncarriers for FA (*p* = 0.048 vs 0.06).

Concerning the incidence of AD, the prospective design allowed for the diagnosis of incident cases over 10 years in 3C and 7 years in AMI with a low rate of loss to follow-up. An active screening of dementia cases was performed over the follow-up, and special attention was paid to the quality of the clinical diagnosis. Although the availability of AD biomarkers obtained through lumbar puncture or PET imaging would have enhanced the accuracy of the etiological diagnosis, their cost, accessibility, and acceptability prevent their use in these population-based cohorts.

### Interpretation in light of the literature

#### Associations between the presence of anti-HSV IgG, hippocampal volume, and WM alterations

Regardless of the APOE status, infected subjects with an IgG level in the highest tercile (reflecting more frequent reactivations of the virus [14]) presented lower HVs compared to uninfected subjects. Infected subjects had also more microstructural alterations in the parahippocampal cingulum and fornix, including lower FA, and higher MD and RD, than uninfected subjects. To our knowledge, this is the first study evaluating the association between the presence of anti-HSV IgG and HV and WM integrity in older adults. Previous studies exploring the association between HSV status and neuroimaging markers were conducted on different populations. Among AD patients, studies [23–25] have previously highlighted a positive correlation between the level of anti-HSV IgG and gray matter volumes, including the volumes in the orbitofrontal cortex and temporal poles. In schizophrenic patients, studies [33–35] revealed an association between HSV infection and cortical atrophy, especially over frontal regions and the cingulate cortex. Finally, in five individuals with a history of HSV-1 encephalitis, a neuroimaging evaluation a few years later (median time 4 years) [36] revealed no significant difference in HV or thickness in the contralateral hemisphere compared to 51 age-matched controls, while a reduction in FA and an increase in MD and RD parameters were reported in bundles connecting the medial temporal lobe to other brain areas.

Although white matter alterations can be seen in neurological pathologies other than AD, the fornix and cingulum bundles are connecting tracts of the limbic system that are thought to be altered prematurely in AD processes. Indeed, previous reports have found significant alterations in DTI parameters in the cingulum or fornix among AD patients, patients with mild cognitive impairment (MCI), or cognitively normal adults who developed amnestic MCI, and these alterations were associated with memory decline [2–5].

Moreover, while the interpretation of DTI parameters remains debated [37, 38], alterations of RD are assumed to reflect myelin damage, suggesting a pathophysiological mechanism underlying the association between HSV infection and AD. Notably, the occurrence of demyelination in the central nervous system following HSV infection was also previously reported in murine studies [39, 40].

#### Associations between HSV status and the incidence of AD according to APOE4 status

Our results highlight an association between the presence or the level of anti-HSV IgG and an increased risk of AD among APOE4 carriers while no significant association was found in the whole sample. Previous studies that have not stratified on APOE status report conflicting results regarding the association between the presence of anti-HSV IgG and cognitive decline or incidence of dementia (reported in [22]) while a significant increased risk of episodic memory decline was found among APOE4 carriers within the Betula study [41]. Regardless of the APOE status, other studies have also observed an increased risk of AD in subjects with frequent reactivations, identified either by the presence of IgM [42, 43] or by a high level of IgG [25].

To note, the presence of anti-HSV IgG reflects an infection that occurred decades before serology, excluding a potential reverse causality bias. Regarding anti-HSV IgG levels (assumed to reflect the frequency of viral reactivations since the first contact with the virus and therefore also for decades), the risk of reverse causation also seems low. Nevertheless, for this marker, such bias cannot be fully excluded if early consequences of AD facilitate viral reactivations and consequently increase the anti-HSV IgG level. With this in mind, we performed a sensitivity analysis to assess whether the significant association found between a high anti-HSV IgG level and incidence of AD in APOE4 carriers could be explained by such problems. In the subsample of APOE4 carriers, after excluding subjects who developed an AD dementia within the subsequent 4 years, the associations remain broadly similar (Supplementary Table 3).

#### Interaction between APOE4 and the presence of anti-HSV IgG

Even if the interaction test was not significant at the level of 0.05, the results of the stratified analyses support the existence of an interaction between APOE4 status and the presence of anti-HSV IgG considering both the risk of AD and lower HVs while no interaction was observed for WM integrity. This apparent discrepancy might reflect a differential impact of APOE4 on gray and white matters as suggested in some studies showing that APOE4 carriers did not differ from noncarriers in DTI parameters of the cingulum and fornix bundles [44]. This is consistent with our data where infected APOE4 carriers had lower HVs while they did not differ from noncarriers regarding DTI parameters (data not shown).

The existence of such an interaction has been reported in the literature. Concerning the risk of AD, our observation is in accordance with results from the Betula Swedish cohort [41, 45], whereas such interaction was not observed in the PAQUID cohort [43]. The existence of an interaction between APOE4 and HSV status has previously been discussed in postmortem studies reporting the presence of HSV DNA in brain regions of older adults according to APOE4 status [46–49], but the results remain controversial. More recently, murine studies have also highlighted an increased HSV-1 load in the brain of APOE4 carriers compared to APOE3 carriers [50–52], and a pathophysiological explanation has been proposed: HSV-1 and the ApoE protein compete for the same binding site to enter neurons. ApoE4, competing less efficiently with HSV-1, facilitates the infection of neurons by the virus [53]. Accordingly, APOE4 status is related to an increased risk of cold sores [48, 49, 54] and to the severity of other viral infections (reviewed in [55]).

The existence of such susceptibility factors could explain that not all the HSV-infected individuals develop AD. However, the APOE4 status may not be the only susceptibility factor and it would be interesting to identify other factors: (i) genetic factors as, beyond APOE4, other genetic risk factors of AD are also linked to HSV [19, 20] or involved in the immune response; (ii) factors linked to the virus such as the type of viral strain; or (iii) factors linked to the immune capacities of the host. Such factors could be useful in distinguishing between subjects who will develop clinical consequences of the infection and those who will remain “healthy carriers”.

### Conclusion

While the underlying mechanism remains uncertain and may involve direct action of the virus and/or the immune response against it, our findings suggest an association between HSV-1 infection and markers of AD pathology. These results reinforce the need to further investigate the infectious hypothesis of AD, especially the associated susceptibility factors such as APOE4 and the possibility of preventive treatments.

## Supplementary information

Supplementary information

Supplementary 1

## Data Availability

The data from the AMI and the 3C cohorts used in this study are available only upon request. Interested researchers may contact e3c.coordinatingcenter@gmail.com for access to 3C data or karine.peres@u-bordeaux.fr for access to AMI data. To request codes, please contact the corresponding author.
