# Supplementary material for: Herpes simplex virus, early neuroimaging markers and incidence of Alzheimer’s disease
Source: Transl Psychiatry. 2021 Jul 31;11:414. doi: 10.1038/s41398-021-01532-2 (PMC8325675; doi:10.1038/s41398-021-01532-2)
Supplement: Supplementary file 1 — Supplementary information [file 41398_2021_1532_MOESM1_ESM.docx]

**Supplementary information**

**Supplementary figure legend**

*Supplementary Figure 1: Flow chart of participants*

Abbreviations: 3C= Three-City cohort, AMI= Aging Multidisciplinary Investigation cohort, HSV=herpes simplex virus, MRI= magnetic resonance imaging

**Supplementary tables**

*Supplementary Table 1: Characteristics of participants included in analysis on incidence of AD according to IgG status. 3C and AMI cohorts.*

|  | | **Sample for incidence of AD analyses (N=1599)**  **N (%)** | **IgG - (N=272)**  **N (%)** | **IgG +**  **(N=1327)**  **N (%)** |
| --- | --- | --- | --- | --- |
| Age | Mean (standard deviation) | 76.7 (5.3) | 76.2 (5.1) | 76.8 (5.4) |
|  | Minimum / Maximum | 65.7 / 96.1 | 66.0 / 96.1 | 65.7 / 92.2 |
| Sex | Male | 716 (44.8) | 138 (50.7) | 578 (43.6) |
| Level of education | Elementary school without diploma | 361 (22.6) | 33 (12.1) | 328 (24.7) |
|  | Short secondary school | 787 (49.2) | 124 (45.6) | 663 (50.0) |
|  | Higher levels | 451 (28.2) | 115 (42.3) | 336 (25.3) |
| Marital status^1^ | Married | 934 (58.5) | 182 (66.9) | 752 (56.8) |
|  | Widowed | 470 (29.4) | 61 (22.4) | 409 (30.9) |
|  | Single or divorced or separate | 192 (12.0) | 29 (10.7) | 163 (12.3) |
| APOE4^1^ | At least one allele | 271 (17.5) | 49 (18.8) | 222 (17.3) |
| Hypertension^1,2^ | Presence | 1266 (79.3) | 209 (76.8) | 1057 (79.8) |
| Diabetes^1,2^ | Presence | 199 (12.5) | 32 (11.9) | 167 (12.6) |
| Hypercholesterolemia^1,2^ | Presence | 933 (58.4) | 158 (58.1) | 775 (58.4) |
| Tobacco consumption^1^ | Former or current smoker | 555 (34.8) | 111 (40.8) | 444 (33.6) |
| Abbreviations: AD Alzheimer’s disease  ^1^ The number of missing data were 3 for marital status, 54 for APOE4, 3 for hypertension, 8 for diabetes, 1 for hypercholesterolemia and 6 for tobacco consumption.  ^2^.Hypertension was defined as taking antihypertensive treatment or having blood pressure ≥ 140/90 mmHg (vs blood pressure <140/90 mmHg without antihypertensive treatment). Diabetes was defined as taking anti-diabetic treatment or having fasting blood sugar ≥ 7 mmol/L or ≥ 11 in case of non-fasting blood sugar (vs fasting blood sugar < 7 mmol/L or <6.1 in case of non-fasting blood sugar and without anti-diabetic treatment). Hypercholesterolemia was defined as taking lipid-lowering treatment or having cholesterol level ≥ 6.2 mmol/L (vs cholesterol level <6.2 mmol/L without lipid-lowering treatment). | | | | |

***Supplementary Table 2: Comparison of the characteristics of the subjects with both HSV status and imaging data versus subjects without these data. 3C and AMI cohorts.***

|  | | **Subjects with data on HSV status and hippocampal volume (N=349)** | **Subjects without these data (N=2221)** | **Subjects with data on HSV status and white matter integrity (N=259)** | **Subjects without these data (N=2311)** |
| --- | --- | --- | --- | --- | --- |
| Age | Mean (standard deviation) | 74.1 (4.5) | 78.0 (5.8) | 73.9 (4.3) | 77.9 (5.8) |
| Sex | Men | 163 (46.7%) | 1040 (46.8%) | 119 (45.9%) | 1084 (46.9%) |
|  | Women | 186 (53.3%) | 1181 (53.2%) | 140 (54.1%) | 1227 (53.1%) |
| Level of education | Missing data | 0 (0.0%) | 6 (0.3%) | 0 (0.0%) | 6 (0.3%) |
|  | Elementary school without diploma | 70 (20.1%) | 633 (28.6%) | 51 (19.7%) | 652 (28.3%) |
|  | Short secondary school | 165 (47.3%) | 1038 (46.9%) | 118 (45.6%) | 1085 (47.1%) |
|  | Higher levels | 114 (32.7%) | 544 (24.6%) | 90 (34.7%) | 568 (24.6%) |
| Marital status | Missing data | 1 (0.3%) | 8 (0.4%) | 0 (0.0%) | 9 (0.4%) |
|  | Married | 232 (66.7%) | 1226 (55.4%) | 174 (67.2%) | 1284 (55.8%) |
|  | Widowed | 79 (22.7%) | 714 (32.3%) | 57 (22.0%) | 736 (32.0%) |
|  | Single or divorced or separate | 37 (10.6%) | 273 (12.3%) | 28 (10.8%) | 282 (12.3%) |
| ApoE4 | Missing data | 18 (5.2%) | 497 (22.4%) | 15 (5.8%) | 500 (21.6%) |
|  | No allele | 262 (79.2%) | 1407 (81.6%) | 193 (79.1%) | 1476 (81.5%) |
|  | At least one allele | 69 (20.8%) | 317 (18.4%) | 51 (20.9%) | 335 (18.5%) |
| Hypertension | Missing data | 1 (0.3%) | 39 (1.8%) | 1 (0.4%) | 39 (1.7%) |
|  | Blood pressure <140/90 mmHg without an antihypertensive treatment | 90 (25.9%) | 400 (18.3%) | 72 (27.9%) | 418 (18.4%) |
|  | Taking an antihypertensive treatment or blood pressure >= 140/90 mmHg | 258 (74.1%) | 1782 (81.7%) | 186 (72.1%) | 1854 (81.6%) |
| Diabetes | Missing data | 0 (0.0%) | 495 (22.3%) | 0 (0.0%) | 495 (21.4%) |
|  | Fasting blood sugar < 7 mmol/L (or <6.1 in case of non fasting blood sugar) without an anti-diabetic | 323 (92.6%) | 1396 (80.9%) | 242 (93.4%) | 1477 (81.3%) |
|  | Taking an anti-diabetic treatment or blood sugar >= 7 mmol/L (or >= 11 in case of non fasting blood | 26 (7.4%) | 330 (19.1%) | 17 (6.6%) | 339 (18.7%) |
| Hypercholesterolemia | Missing data | 0 (0.0%) | 359 (16.2%) | 0 (0.0%) | 359 (15.5%) |
|  | Cholesterol level <6.2 mmol/L without a lipid-lowering treatment | 145 (41.5%) | 725 (38.9%) | 104 (40.2%) | 766 (39.2%) |
|  | Taking a lipid-lowering treatment or cholesterol level >= 6.2 mmol/L | 204 (58.5%) | 1137 ( 61.1%) | 155 (59.8%) | 1186 (60.8%) |
| Tobacco consumption | Missing data | 0 (0.0%) | 102 (4.6%) | 0 (0.0%) | 102 (4.4%) |
|  | Non smoker | 228 (65.3%) | 1360 (64.2%) | 168 (64.9%) | 1420 (64.3%) |
|  | Former or current smoker | 121 (34.7%) | 759 (35.8%) | 91 (35.1%) | 789 (35.7%) |

***Supplementary Table 3: Sensitivity analyses. Association between anti-HSV IgG level and the incidence of Alzheimer’s disease in APOE4 carriers after excluding AD cases occurring within the first four years – Adjusted Cox models. 3C and AMI cohorts.***

|  |  |  | | | |  |
| --- | --- | --- | --- | --- | --- | --- |
|  |  |  | APOE4-positive subjects^1^ (n=250 including 33 cases  or n=201 including 29 cases)^2^ | | |  |
|  |  |  | aHR | 95% CI | p-value |  |
| **IgG levels in terciles (in all subjects)**^3^ |  |  |  |  |  |  |
| 1st tercile (vs negative IgG) |  |  | 1.29 | 0.35-4.70 | 0.70 |  |
| 2nd tercile (vs negative IgG) |  |  | 1.60 | 0.46-5.59 | 0.46 |  |
| 3rd tercile (vs negative IgG) |  |  | **3.34** | **1. 08-10.31** | **0.04** |  |
| **IgG levels in terciles (in IgG+ subjects)**^3^ |  |  |  |  |  |  |
| 2nd tercile (vs 1st tercile) |  |  | 1.50 | 0.47-4.85 | 0.50 |  |
| 3rd tercile (vs 1st tercile) |  |  | 2.71 | 0.997-7.36 | 0.05 |  |

Abbreviations: aHR = adjusted hazard ratio, 95% CI = 95% confidence interval

^1^ Adjusted for sex, level of education and cohort.

^2^ Number of subjects for analysis in the whole sample or in the subsample of IgG+ subjects, respectively.

^3^ IgG levels in the 1st tercile were <17.7, in the 2nd tercile were ≥17.7 and <27.9 and in the 3rd tercile were ≥27.9.

***Supplementary Information***

Regarding HSV serologies, we followed the threshold recommendations of the manufacturer: i) an index value ≥1.10 was considered as the presence of anti-HSV IgG (or IgM), ii) an index value < 0.9 was considered as the absence of anti-HSV IgG (or IgM), and iii) no conclusions could be drawn on samples with an index value between 0.9 and 1.10. For the latter, if the index value remained inconclusive after a second test, the corresponding samples were excluded from analysis (n=40).

MRI scans were performed using an ACHIEVA 3T scanner (Philips Medical System, Netherlands) with a SENSE 8-channel head coil. Anatomical high-resolution MRI volumes were acquired in the transverse plane using a 3D MPRAGE T1-weighted sequence with the following parameters: TR=8.2 ms, TE=3.5 ms, 7-degree flip angle, FOV 256x256 mm^2^, 180 slices, no gap and voxel size of 1 mm^3^. Two diffusion-weighted images with opposite polarities were performed using a spin echo single shot EPI sequence with the following parameters: TR=9700ms in 3C and 6770 ms in AMI, TE=60 ms, 90-degree flip angle, FOV 224x224 mm^2^, 60 slices, no gap and voxel size of 2 mm^3^. One b0 image was acquired, and diffusion gradients were applied in 21 noncollinear directions (b-value=1000 s/mm^2^). To increase the signal-to-noise ratio, the sequence was repeated in two successive runs for each polarity. All acquisitions were aligned on the anterior commissure-posterior commissure plane (AC-PC).
