# Supplementary figures and images for: Herpes simplex virus, early neuroimaging markers and incidence of Alzheimer’s disease
Source: Transl Psychiatry. 2021 Jul 31;11:414. doi: 10.1038/s41398-021-01532-2 (PMC8325675; doi:10.1038/s41398-021-01532-2)

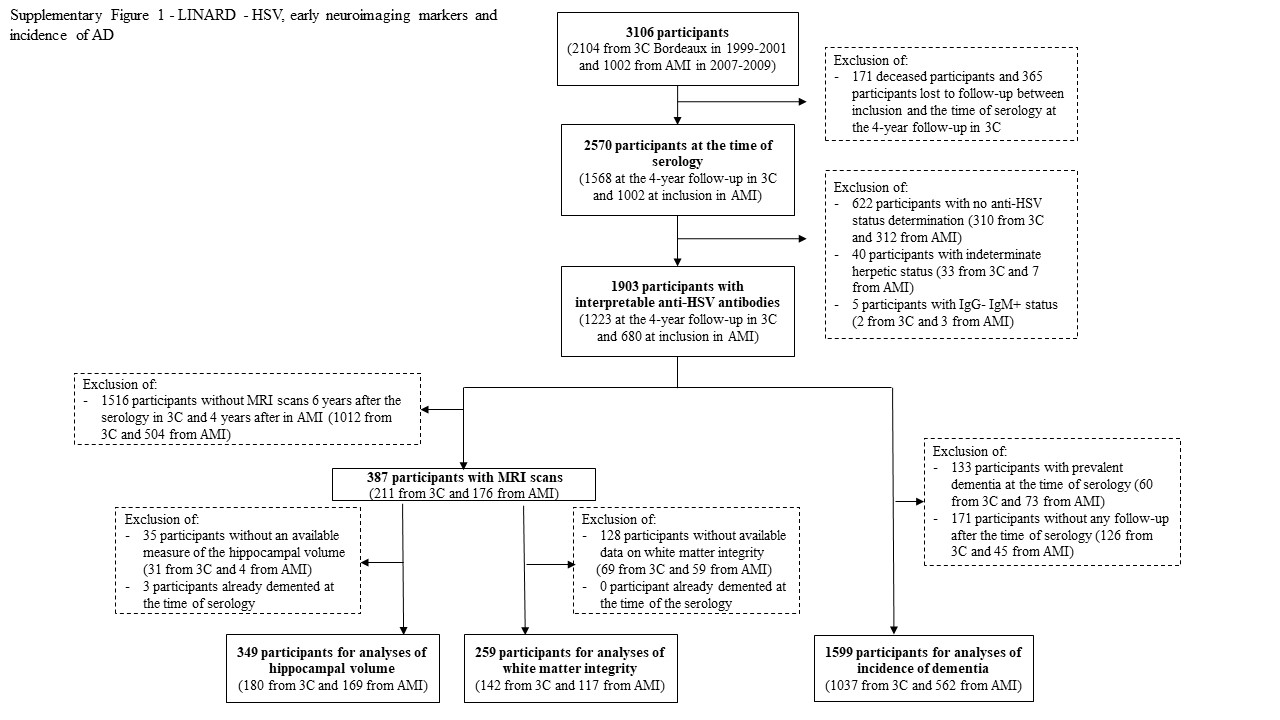

Supplement: Supplementary file 2 — Supplementary 1 [file 41398_2021_1532_MOESM2_ESM.jpg]
